# Advances and development of prostate cancer, treatment, and strategies: A systemic review

**DOI:** 10.3389/fcell.2022.991330

**Published:** 2022-09-09

**Authors:** Sana Belkahla, Insha Nahvi, Supratim Biswas, Irum Nahvi, Nidhal Ben Amor

**Affiliations:** ^1^ Department of Basic Sciences, Preparatory Year Deanship, King Faisal University, Al Hofuf, Saudi Arabia; ^2^ University of Cape Town, Department of Human Biology, Cape Town, South Africa; ^3^ College of Computer Engineering and Science, Prince Mohammad Bin Fahd University, Al Khobar, Saudi Arabia; ^4^ Public Health Department, Veterinary College, King Faisal University, Al Hofuf, Saudi Arabia

**Keywords:** nanotherapeutics, prostate, therapeutic, metastasis, mortality, Saudi Arabia, nanomedications

## Abstract

The most common type of cancer in the present-day world affecting modern-day men after lung cancer is prostate cancer. Prostate cancer remains on the list of top three cancer types claiming the highest number of male lives. An estimated 1.4 million new cases were reported worldwide in 2020. The incidence of prostate cancer is found predominantly in the regions having a high human development index. Despite the fact that considerable success has been achieved in the treatment and management of prostate cancer, it remains a challenge for scientists and clinicians to curve the speedy advancement of the said cancer type. The most common risk factor of prostate cancer is age; men tend to become more vulnerable to prostate cancer as they grow older. Commonly men in the age group of 66 years and above are the most vulnerable population to develop prostate cancer. The gulf countries are not far behind when it came to accounting for the number of individuals falling prey to the deadly cancer type in recent times. There has been a consistent increase in the incidence of prostate cancer in the gulf countries in the past decade. The present review aims at discussing the development, diagnostics *via* machine learning, and implementation of treatment of prostate cancer with a special focus on nanotherapeutics, in the gulf countries.

## 1 Introduction

Prostate cancer (PC) is a type of cancer that occurs in a small male gland called the prostate. The main function of the prostate is to produce the seminal fluid that nourishes and transports sperms. PC is one of the most common cancers among men representing a major public health issue as about one man in six is being diagnosed with PC, but it is highly treatable in its early stages. Prostate cancer is usually identified by a blood test to measure prostate-specific antigen levels (PAS), (PSA > 4 ng/ml), a glycoprotein normally expressed by prostate tissue and/or digital rectal examination (Rebello et al., 2021).

Prostate cancer is the second most frequent disease in the world, according to the World Health Organization (WHO) with 1,414,259 new cases around the world, and is the fifth leading cause of male cancer-related deaths. According to Globacan 2020 estimates, it caused 375,304 cases of deaths among men worldwide of all ages (Global Cancer Statistics 2020). In the United States, Africa, and Europe, PC has been found to be the leading cause of death after lung cancer. In Saudi Arabia, the International Agency for Research on Cancer (IARC) estimated that the number of incident cases was 693, the age-standardized incidence rate (ASIR) for prostate cancer was 7 per 100,000 men in 2020, and the age-standardized mortality rate (ASMR) was 2.5 per 100,000 men (Panigrahi et al., 2019). The age-standardized rate (ASR) of PC in Arab countries is relatively low compared to Europe and North America; it may be due to several factors such as the low prostate-specific antigen (PSA) test screening and specific biological differences among Arab men (Osman et al., 2018).

Obesity, unhealthy diet, tobacco and alcohol consumption, family history, racial differences, and age are some of the potential risk factors linked with PC (Perdana et al., 2016). According to the National Cancer Institute (NCI), two-thirds of patients diagnosed with PC are either 65 years old or more than that. The average age range during diagnosis is between 60 and 70 years (Siegel et al., 2018).

The purpose of our study is to evaluate and analyze the prostate cancer rate in Saudi Arabia and other Arab countries. We interpreted data from different studies and articles published in the local medical literature worldwide, especially in Saudi Arabia.

## 2 Epidemiology

### 2.1 Incidence of prostate cancer worldwide

Based on the International Agency for Research on Cancer 2020 estimates, we have evaluated the incidence and mortality rates of prostate cancer worldwide and in the Arab population taking Saudi Arabia as an example.

Based on Globacan, in 2020, 1,414,259 new cases of prostate cancer were registered worldwide representing around 7.2% of all cancers in men. The estimated number of new cases of prostate cancer is highly variable worldwide (Table1). Europe and Asia have the higher number of new cases 93,173, and 22,421, respectively, with an estimated incidence rate of 33.5% and 26.5%, respectively, compared to non-developed countries such as Africa and Oceania with an incidence rate of 6.6% and 1.6%, respectively (Table 1).

**TABLE 1:** Incidence of prostate cancer worldwide.

Population	Number of new cases	Incidence rate (%)	Number of deaths
Europe	473,344	33.5	108,088
Asia	371,225	26.2	120,593
North America	239,574	16.9	37,192
Latin America and Caribbean	214,522	15.2	57,415
Africa	93,173	6.6	47,249
Oceania	22,421	1.6	4,767
Total	1,414,259		

Differences in the number of new cases vary extremely between the populations at the highest rate (Germany, 67,959 cases) and the populations with the lowest rate (Bhutan, three cases). The reason for these differences among populations is not entirely clear.

According to a recent statistical analysis, the worldwide variations in prostate cancer incidence might be the result of overscreening in developed countries. It was shown that around 20%–40% of the prostate cancer cases in the United States and Europe were identified by PSA testing.

**FIGURE 1:**
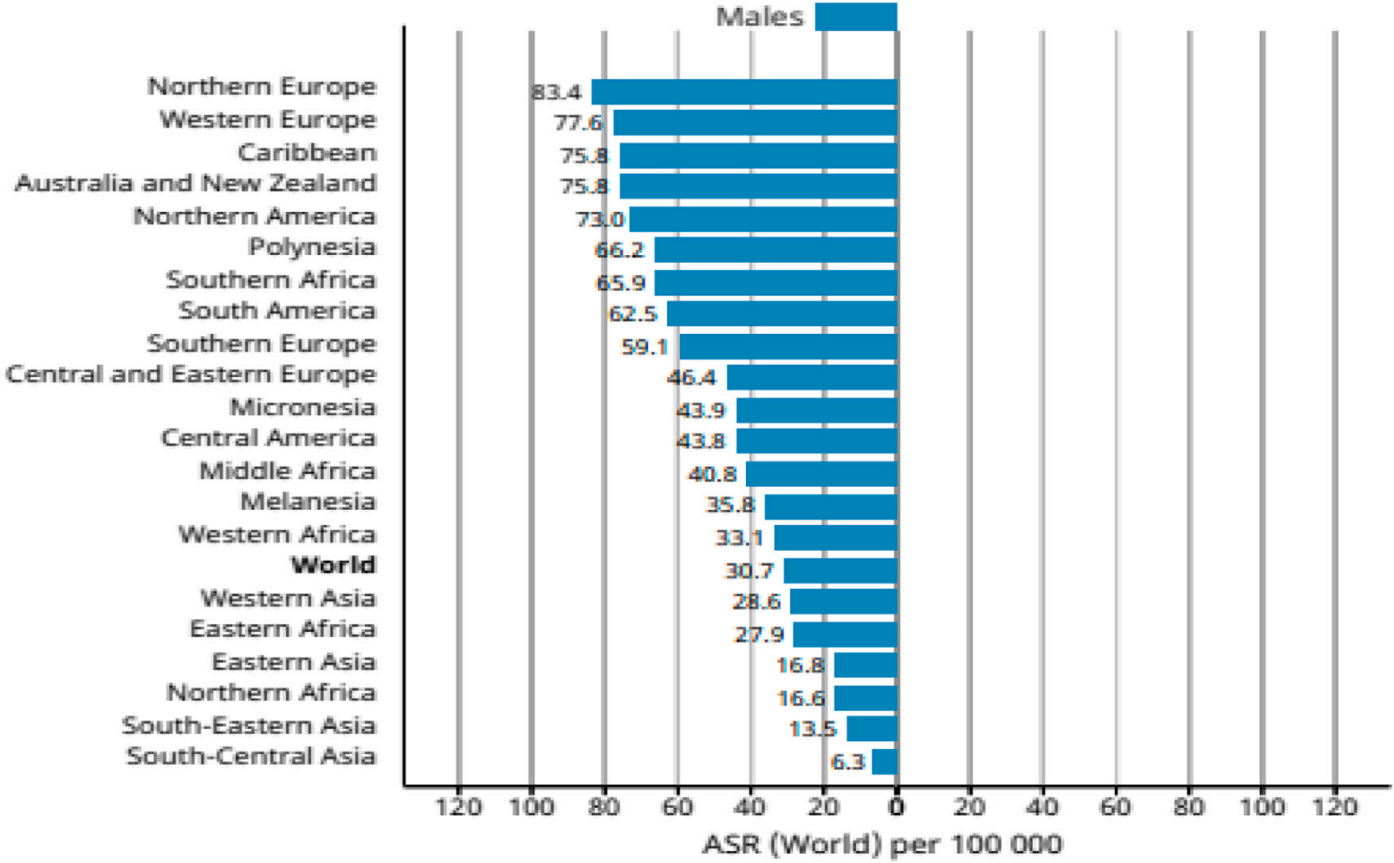
Association of obesity with different types of cancers.

Generally, the rate of incidence of cancer increases with age, the age-standardized rate of patients with prostate cancer (ASR) was highest in Northern and Western Europe (83.4 and 77.6 per 100,000 people, respectively), followed by the Caribbean (75.8 per 100,000 people) and North America (73.0 per 100,000 people) (Figure 1).

Based on new statistics, about 1 man in 41 died of prostate cancer, which led to 375,304 (3.8% of all cancer) deaths globally in 2020 (9,958,133 deaths). Asia has the highest number of deaths (120,593), followed by Europe (108,088). However, the number of death cases in Oceania is lower than that in developed countries with 4,767 deaths (Table 1).

### 2.2 Distribution of cases and deaths by region among Arab countries

Prostate cancer incidence is lower in Arab countries than in Canada, Germany, and the United States, where extensive epidemiological studies are easier to conduct. In the United States, prostate cancer is the second most common cancer accounting for 12.5% of cases (209,512 new cases) of all new cancers registered in 2020 (1,674,081 cases). In Saudi Arabia, the number of new cases of PC was 693 (2.5% of all cancers) with the crude rate and age-standardized incidence rate being 3.4 and 7.0 per 100,000, respectively, compared to other Arab countries. Qatar has the lowest number of new cases (104) with an ASIR of 21.1 per 100,000, followed by Oman and Kuwait with 186 and 255 new cases, respectively, and ASIRs were 13.8 and 19.6 per 100,000, respectively.

However, the Arab populations in North Africa have a higher number of new cases than the Middle East Arab population. Egypt and Morocco have the highest incidence of new cases with 4,767 and 4,429, respectively, and ASIRs were 13.9 and 23.6 per 100,000, respectively.

The mortality rate in Middle Eastern, North African, and Asian men was found to have a lower prevalence of prostate cancer than Europe, America, and Canada (Table 2).

**TABLE 2:** Mortality rate among Middle East countries and Canada, America, and Europe.

Country	Number of new cases	ASIR	Mortality
Tunisia	1,186	17	541
Algeria	3,597	17	1,635
Morocco	4,429	23.6	1875
Egypt	4,767	13.9	2,227
IRAQ	1,117	12.9	416
Jordan	1,568	15.8	142
Kuwait	255	19.6	52
Lebanon	1,029	28.5	360
Oman	186	13.8	73
Qatar	104	21.1	18
Saudi Arabia	693	7.0	204
Canada	29,972	80.4	4,744
United States	209,512	72.0	32,438
Germany	67,697	66.0	15,507
World	1,414,259	30.7	375,304

As of 2020, the estimated number of deaths in Saudi Arabia is 204, which is higher than that in Qatar (18 death cases), Kuwait (52 deaths), Oman (73 deaths), and Jordan (142 deaths). However, the ASIR in Saudi Arabia (2.5 per 100.000) is lower than that in Qatar and Jordan with 4.8 and 5.3 per 100.000, respectively. Recent diagnosis of the incidence of prostate cancer worldwide has shown that African–American men have the highest incidence and are more susceptible to developing the disease at an early age compared to other racial and ethnic groups. This result is confirmed not only for African–American men but also for Caribbeans and Black men in Europe. Chu et al. stated that the incidence rate of PC was 40 times higher among African–American men than those in Africa supporting the main hypothesis that the incidence rate of cancer can be related to the genetic background of populations.

In Saudi Arabia, prostate cancer is the major cause of morbidity and mortality in males within the age group of 50–70 years. According to Saudi cancer registry reports, the ASR varies across Saudi regions. The Eastern province and Riyadh have the highest male ASR mean with 9.6 and 7.2 per 100,000, respectively. This is followed by the Makkah region and ASIR region with 5.9 and 4.9 per 100,000 patients. Conversely, in Jazan and Hail regions, the ASR average is 1.8 per 100.000 men, which is the lowest ASR average compared to other regions (Ali A et al., 2018). Based on the grade, localization, and size of the tumor, scientists have identified five types of prostate cancer. More than 95% of prostate cancers are adenocarcinomas and up to 5% of prostate cancers may be carcinomas, neuroendocrine tumors, prostate sarcomas, or transitional cell carcinomas.

Based on the Saudi registry of cancer (Bandar, 2020), the most common PC subtype over 22 years (from 1994 to 2016) was adenocarcinoma (88% of the total cases), followed by carcinoma and sarcomas with 5% and 4% of the total cases.

Prostate cancer, like other types of cancer, does not have an exact cause. In fact, several risk factors may be involved, including genetic mutation, alteration in lipid metabolism, human papillomavirus infection (HPV), and racial differences (Al-Abdin OZ et al., 2013; Ross-Adams H et al., 2015; Travis et al., 2016; Siegel et al., 2018).

### 2.3 Potential risk factors of prostate cancer

The potential risk factors of prostate cancer can be divided into non-modifiable factors such as age, race, and family history and modifiable risk factors such as diet, physical activity, smoking, and obesity (Perdana et al., 2016).

#### 2.3.1 Non-modifiable risk factors

##### 2.3.1.1 Race/ethnicity

Several recent studies suggest that race and ethnicity are considered as essential risk factors for PC (Wu et al., 2012). According to the latest statistics reports, the incidence and mortality rates of PC remain high among African–American men, West African ancestry from the Caribbean, and South American men than white men (Globacan). However, the lowest incidence of PC is essentially found in Middle Eastern, North African, and Asian men (Akaza H et al., 2011; Powell et al., 2013). Data from the National Cancer Institute has shown that African–American men usually have the highest rate (1 in 6 men) compared to other ethnic races such as non-Hispanic White (NHW) men who have a risk of 1 in 8 men being diagnosed with PC in their lifetime. Recent evidence suggests that the difference in the incidence and mortality rate is multifactorial. Comparing the genetic and transcriptome profiles of 596 African–American men and 556 NHW men with PC from different races, researchers (De Santis et al., 2016) suggest that genes controlling the inflammatory pathways (e.g., CCL4, IFNG, CD3, IL33, and ICOSLG) are upregulated in African–American men with downregulation of DNA repair genes (e.g., MSH6 and MSH2) which are highly expressed in non-Hispanic white (NHW) repair pathway (PTEN) deletions (11.5% in African-American vs. 30.2% in NHW) and metabolic pathways involving glycolysis and cell cycle activity (Nair Sujit S et al., 2022).

Genome-wide association studies (GWAS) are revolutionary studies used over the past decade in genetic research to associate the genetic variation with common diseases or traits in a population (Jabril et al., 2021). Darst et al. (2020) stated that a specific variant (rs72725854) at locus 8q24 was associated with the high frequency of prostate cancer in African–American men, African descent men in Caribbean nations, and West Africans. However, there was no genetic association in populations with European ancestry.

##### 2.3.1.2 Age

Prostate cancer risk may grow with age. Indeed, older men are more prone to get PC than younger men (under 40), who have a lower risk of being diagnosed with the disease (Howlader et al., 2013). The risk of prostate cancer rises rapidly after age 50, and according to analytic studies, about 6 in 10 cases of prostate cancer are found in men older than 65 who have lower overall survival. As a result, it is highly recommended to encourage older men (over 60) to get prostate-specific antigen (PSA) test screening frequently (Vickers et al., 2010; Jayadevappa et al., 2011).

##### 2.3.1.3 Family history

In addition to age and race, family history is one of the nonmodifiable risk factors for prostate cancer in men (Addo et al., 2010). Zheng et al. have examined five single-nucleotide polymorphisms (SNPs) and have found a significant association with PC with a family history of PC (Jun et al., 2018). Gayathri Sridhar et al. (2010) demonstrated that the risk of prostate cancer increased for men with a family history of any cancer or prostate cancer in first-degree relatives (OR = 2.68, 95% CI = 1.53–4.69) and parents alone (OR = 2.68, 95% CI = 1.53–4.69). The Health Professional Follow-Up Study (HPFS) followed up more than 3,695 patients for 18 years (1986–2004) and confirmed a 2.3-fold increased risk of PC with a family history of PC (95% confidence interval (CI) = 1.76–3.12). Several other studies confirmed that family history is the most important risk factor compared to age and ethnicity in the development of prostate cancer (Maria et al., 2021). To date mismatch repair (MMR) genes (MLH1, MSH2, MSH6, and PMS2) and homologous recombination genes (BRCA1/2, ATM, PALB2, and CHEK2) are potentially associated with PC.

#### 2.3.2 Modifiable risk factors

##### 2.3.2.1 Obesity

**FIGURE 2:**
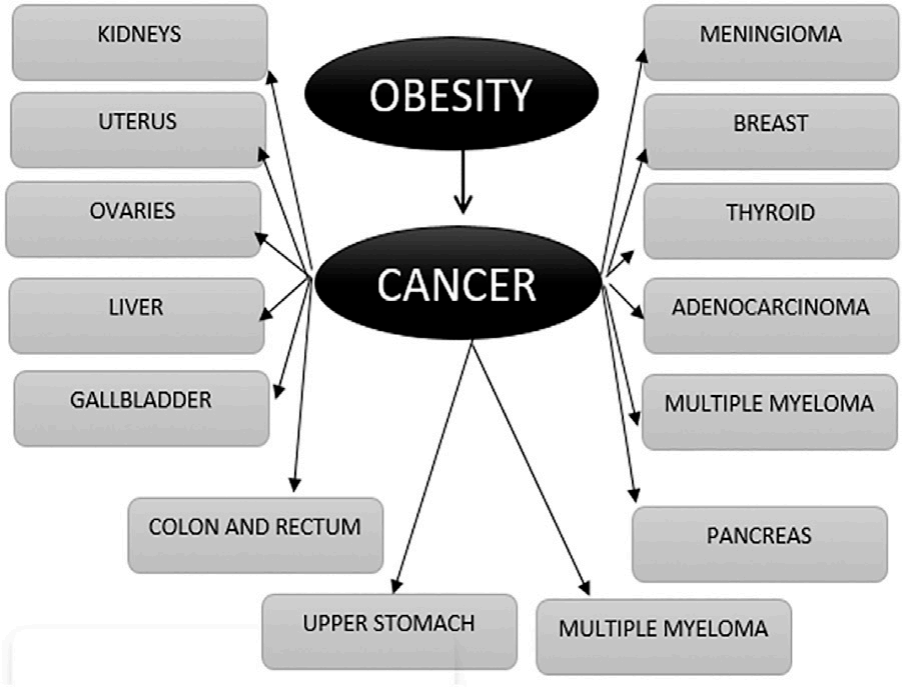
Steps of the approach using the feature selection technique “CIWO”.

Overweight and obesity are complex diseases involving an excessive amount of body fat. Recent studies confirm that obesity is a serious public health issue and is associated with at least 13 different types of cancers, namely, multiple myeloma (Susanna et al., 2007), meningioma (Chuan et al., 2014), uterus (Hao et al., 2021), breast (Kyuwan et al., 2019), thyroid (Theodora et al., 2014), ovary (Mauricio et al., 2018), liver (Carlo et al., 2019), adenocarcinoma (Katherine et al., 2020), gallbladder (Larsson SC and Wolk A 2007), colon, rectum (Pavel et al., 2018), pancreatic (Prashanth et al., 2019), and upper stomach cancers (Jacek et al., 2019) (Figure 2). Recently, three meta-analyses have confirmed a positive correlation between overweight and prostate cancer incidence (Boeing et al., 2013; Meng-Bo Hu et al., 2014).

A recent study in Saudi Arabia, including 81 patients in Arar Hospital, demonstrated a significant relationship between prostate cancer and obesity as 62.5% of cases were obese and 37.5% were nonobese (*p* < 0.05) (Abdullah et al., 2017). In contrast, Abdulaziz A et al. (2019) stated that there is no association between obesity (BMI ≥ 30) and the incidence of prostate cancer (relative risk = 1.05: 95% CI: 0.51–2.14) in a case-control study that included 2,160 male patients in Saudi Arabia.

##### 2.3.2.2 Smoking and alcohol intake

As Saudi Arabia is an Islamic country, according to Islamic laws, alcohol consumption is completely banned in Saudi Arabia. Thus, the Saudi population is considered to have the lowest alcohol consumption worldwide. In contrast, smoking seems to be a prevalent habit because cigarette smoking was reported by 21.4% among the Saudi population (Aljoharah M et al., 2018). According to the International Agency for Research on Cancer (IARC), more than 60% of chemicals that are present in a cigarette are carcinogens of class I and class II. Cigarette smoke contains more than 4,000 different chemicals such as polycyclic aromatic hydrocarbons (PAHs) (Huncharek et al., 2010) which are hydrocarbons highly linked to skin, lung, bladder, liver, and stomach cancers. PAHs can be genotoxic and bind to DNA-inducing mutations that enhance cancer proliferation, or it can be nongenotoxic and promote cancer evasion and progression (Baird W.M et al., 2005). Therefore, functional polymorphisms in genes involved in PAH metabolism and detoxification may modify the effect of smoking on PC (Noor et al., 2016). An association with smoking could also have a hormonal basis: male smokers were found to have elevated levels of circulating androsterone and testosterone, which may increase PC risk or contribute to cancer progression (Huncharek et al., 2010; Li J et al., 2012). A meta-analysis published by Michael Huncharek et al. (2010) evaluated the relationship between smoking and PC and indicated that current smokers had a 24%–30% greater risk of fatal PC. These observational cohort studies enrolled 21,579 PC patients and confirmed the association of smoking with PC recurrence and mortality. In addition, Gutt et al. (2010) examined 434 patients, and they stated that current smokers and former smoker patients have a recurrence rate up to 5.2 and 2.9 times greater, respectively, than the rate of life-long non-smokers.

##### 2.3.2.3 Diet

###### 2.3.2.3.1 Animal fat

Lipids are macromolecules responsible for storing energy, signaling, and acting as structural components of cell membranes. Lipids are divided into two groups: fats and steroids. A high-fat diet has been linked to an increased risk of prostate, breast, and colon cancers, among other malignancies (Bianka et al., 2020). Le Marchand et al., Mucci et al., Platz et al., and Pauwels et al. confirmed a positive correlation between animal fat consumption and high incidence and mortality of PC patients (Pauwels.,2011).

The World Research Cancer and Fund (WCRF) study showed that consumption of <500 g of red meat per week (OR = 0.77, 95% CI: 0.61, 0.98) was negatively correlated with a high risk of PC. Several other studies evaluate the relationship between red meat consumption and PC. They indicated that men receiving more than five serving parts of meat per week had a higher risk to develop PC than men who eat less than 1 serving/week (Aronson WJ et al., 2010). It is still not clear what are the underlying possible biological mechanisms between high-fat dietary intake and PC risks, but it is possible that high energy intake increases basal metabolism and enhances prostate carcinogenesis *via* androgen. (Schultz C et al., 2011; Arab L et al., 2013).

###### 2.3.2.3.2 Calcium and milk

As milk and dairy products are rich in fat and calcium, they may be involved in the process of tumorigenesis. A meta-analysis of 12 publications confirmed a significant correlation between the high dairy intake of milk and calcium (>2000 mg/day) and advanced-stage and high-grade prostate cancer (Schultz C et al., 2011). Calcium plays a key role in prostate carcinogenesis by controlling PC cell growth and apoptosis (Kathryn MW et al., 2015). The biological pathways in which calcium can alter prostate carcinogenesis are still not clear. However, it was shown that intracellular calcium pools can control PC cell growth and significantly decrease their susceptibility to apoptosis (Kathryn MW et al., 2015).

## 3 Prostate cancer detection reviewed in Gulf regions

It is important to point out cancer in an early stage for the health of the patient and research society. Early detection of cancer makes treatment tremendously coherent. It can cause death because of poor diagnosis. Although tissue biopsy is the standard for diagnosis, the classification and recognition have improved *via* imaging and indicators or biomarkers (Litwin et al., 2017). In this review, we have only explained in detail the detection of PC *via* machine learning as the conventional diagnostics have been explained previously in other research articles in detail.

By integrating machine learning and artificial intelligence in the research of prostate cancer early detection, thepositive and accurate result has increased. However, there are many challenges in the investigation process due to extremely high and low dataset samples. To improve the detection rate, the best feasible solution can be found using meta-heuristic algorithms.

**FIGURE 3:**
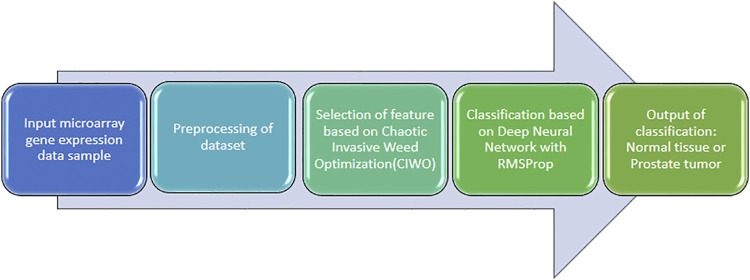
Deep learning methods such as long short-term memory (LSTM) and Residual Net (ResNet-101).

One of the approaches is by using a deep learning model for the detection of prostate cancer (AIFSDL-PCD) using a microarray gene expression dataset (Figure 3). This approach used a feature selection technique “chaotic invasive weed optimization (CIWO)” after preprocessing data samples. Furthermore, a deep neural network (DNN) model with the RMSProp optimizer can be involved in prostate cancer detection. The output classification, as well as the analytical complicacy, is improved to a greater extent (Abdul rhman M. et al., 2022).

**FIGURE 4:**
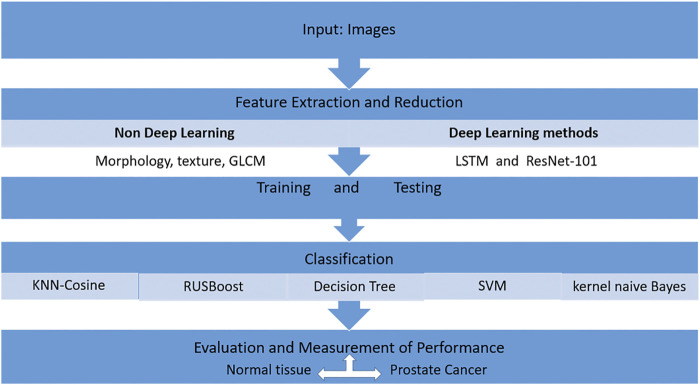
Model using the correlation feature selection method and random committee model (RC).

Traditional techniques such as support vector machine (SVM), decision tree (DT), k-nearest neighbor-cosine (KNN-Cosine), RUSBoost tree, and kernel naive Bayes are unable to extricate the complicated characteristics of cancer. Other deep learning methods such as long short-term memory (LSTM) and Residual Net (ResNet-101) could be used as a better predictor for the detection of prostate cancer (Figure 4). This approach compares the output of the deep learning methods and non-deep learning methods with the traditional feature extraction approach. First, the texture is extracted from a non-deep learning approach, and then, machine learning techniques are applied. The classification techniques are used, such as SVM, to detect prostate cancer. The highest detection is given by ResNet-101 based on its Rectified Linear Unit (ReLU) function and an optimized gradient descent algorithm (Saqib I et al., 2021).

**FIGURE 5:**
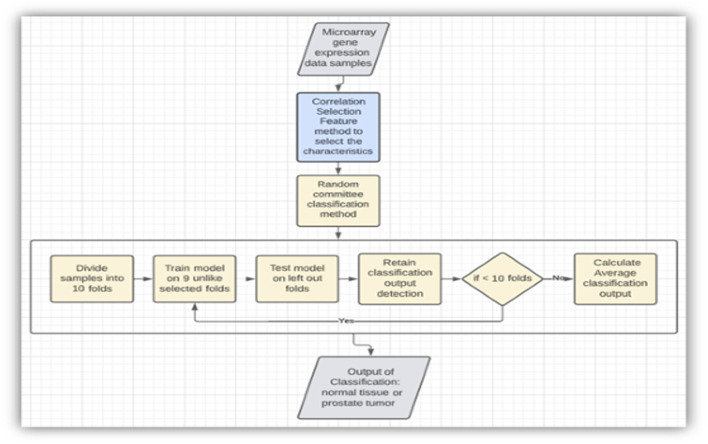
Age-standardized rate of patients with prostate cancer worldwide.

As mentioned, to increase the rate of correctness and precision of detection, appropriate machine learning techniques are utilized. In one of the suggested approaches, as in Figure 5, the input captured is a microarray dataset. By using the correlation feature selection method, the appropriate characteristics are selected. Then, by applying a random committee model (RC) to the input data, a few experiments are conducted using a 10-fold cross-validation technique for the evaluation of this current approach. This revealed a higher accuracy rate of output and increased the implementation time (Abdu et al., 2021).

Experimental output is announced using some evaluation metrics such as the confusion matrix, precision, recall, specificity, F1-Score, and accuracy to show the efficiency of the proposed technique for prostate cancer detection. Also, the comparison results between these evaluation matrices confirmed the accuracy result as opposed to using all features.

## 4 Treatment of prostate cancer

Finalizing a treatment regime for the prostate cancer patient is determined keeping into consideration factors like the rate at which the cancer is growing, whether metastasis has set in, and most importantly implicated benefits and side effects of the treatment methods to be undertaken. The choice of the treatment procedure also considers the factor of risk of death from other causes. The conventional therapies for prostate cancer are chemotherapy and novel hormone therapies.

Different conventional therapies for prostate cancer are

4.1 Chemotherapy
4.2 Novel hormone therapies

### 4.1 Chemotherapy

The chemotherapeutic route of treatment involves the usage of drugs like docetaxel, cabaxitaxel, and mitoxantrone. Docetaxel has been one of the most successful drugs to improve the overall survival in metastatic castration-resistant prostate cancer. Improvement in overall survival symptoms, prostate-specific antigen, and quality of life was seen in metastatic castration-resistant prostate cancer patients when treated with docetaxel and prednisolone. Typically, docetaxel is administered intravenously every 3 weeks for recommended 10 cycles (Litwin et al., 2017; He MH et al., 2018). The mode of action, although not fully understood but was observed to have targeted microtubules during mitosis and interphase, caused stabilization of the mitotic spindle leading to mitotic and cell proliferation arrest causing cell death. Cabaxitaxel is another approved chemotherapeutic drug, indicated for usage in the treatment of prostate cancer. Cabaxitaxel has got a similar mode of action to docetaxel where it disrupts microtubule function causing cell death. Cabaxitaxel is typically administered intravenously once every 3 weeks. Cabaxitaxel is also recommended for a 10-cycle regime, keeping in view the condition of the patient (Qin et al., 2017).

### 4.2 Novel hormone therapy

The most widely used treatment route in treating metastatic prostate cancer was the process of castration which was followed for nearly a century. The treatment management involving castration came up with a success rate of 60%–70% depending on different criteria. A decrease in the success rate was observed with an increase in the secretion of adrenal androgen hormones in association with the evolution of upregulated or mutated androgen receptors (Qin W et al., 2017; Omabe K et al., 2021). At the developmental stage, prostate cancer relies on the androgenic hormones to proliferate. A logical route to arresting the progression of prostate cancer is by lowering the levels of androgen hormones or blocking the androgenic action. The different types of hormonal therapy for prostate cancer are

a. Bilateral orchiectomy: nearly a century ago, this mode of treatment for prostate cancer was introduced involving the removal of both the testicles. This is quite a systematic way of treatment as it removes the source of testosterone production (Litwin et al., 2017).
b. LHRH agonists: LHRH agonist or luteinizing hormone-releasing hormone agonist are medications that restrict the testicles from producing testosterone. The only difference between the LHRH agonist treatment method with orchiectomy is that the effects of LHRH agonists are found to be reversible, reliving the testosterone production soon after the treatment stops.
c. LHRH antagonists: LHRH antagonists are a class of drugs, known as gonadotropin-releasing hormone (GnRH) antagonists, which initiate the inhibition of the testosterone-like LHRH agonists by the testicles at a much faster pace devoid of the flare caused by the LHRH agonists. The FDA has approved an injectable drug called degarelix (Firmagon), administered monthly, for the treatment of advanced prostate cancer; on the contrary, it may cause severe allergic reactions. The FDA has also approved an oral LHRH antagonist, known as relugolix (Orgovyx), for the treatment of advanced prostate cancer.
d. Androgen synthesis inhibitors: there are other parts of the body like the adrenal gland other than the testicles which produce testosterone that can fuel the prostate cancer cells. Thus, androgen synthesis inhibitors are molecules that target an enzyme called CYP17 and resist cells from synthesizing testosterone. Examples of androgen synthesis inhibitors are abiraterone acetate and ketoconazole (Litwin et al., 2017).

## 5 Role of nanoparticles in prostate cancer treatment

The conventional way of treating prostate cancer faced several challenges like depleted accumulation levels, faster clearance, or drug resistance at the tumor site. These factors led to the decline in effects of the chemotherapeutic drugs. Problems like decreased bioavailability and nonspecific distribution with severe side effects in delivery of anticancer drugs have been resolved by nanomedications like metallic nanoparticles and liposomes etc., that improve the therapeutic index with reduced non-specific distribution and higher dose of drugs (Sanna V et al., 2012). The world of nanotechnology has opened a new avenue for the more advanced treatment of prostate cancer. Nanoparticles are remarkably efficient carriers of different therapeutic biomolecules by virtue of their unique size, large surface-to-volume ratio drug encapsulation capability, and modifiable surface chemistry (Thakur A et al., 2021). For decades, common chemotherapeutic agents, like docetaxel and paclitaxel, were employed for the treatment of prostate cancer, which had disadvantages like non-selectivity toward cancerous tissues, thus causing injuries to the surrounding normal tissues, resulting in the lower therapeutic index and increased drug resistance. These were initial motivations behind developing newer ways of addressing an age-old problem (Qin W et al., 2017). Nanotechnology emerges as a boon as it has the potential to address numerous issues that hinder the success of cancer therapy. Some of the issues that get better solutions by the application of nanoparticles are 1) enhanced delivery of poorly water-soluble drugs, 2) site-directed delivery of drugs toward specific biological and molecular targets, 3) development of innovative diagnostic tools, and 4) combination of therapeutic agents with diagnostic probes (Thakur et al., 2021). Nanoparticle-facilitated molecular targeted therapy for cancer is a strategy of considerable promise which received astounding success in minimizing non-specific biodistribution and therapeutic index of traditional chemotherapeutic drugs. In recent times, prostate-specific membrane antigen (PSMA)-conjugated nanoparticles have proved to be a potent management option for prostate cancer. This gives rise to better selectivity of drugs so that only diseased cells are targeted rather than normal ones (Cherian AM et al., 2014). Epigallocatechin 3-gallate (EGCG), a natural product isolated from green tea, has been observed to possess potential chemo-preventive effects in both *in vivo* and *in vitro* models of prostate cancer. Modern cancer treatment approaches have now been catered to by the introduction of nanoparticle-mediated targeted drugs (Litwin et al., 2017; Thakur et al., 2021). To put this in perspective, utilization of nanographene oxide (NGO) has increased many folds over the past decades since the quality of both side’s graphitic domains persists for drug loading due to the presence of hydrophobic interaction, π-π staking, and hydrogen bonding in their structure. Nanotechnology has been put to test for decades now to study treatment procedures for different kinds of cancer. The main focuses which help the popularity of nanotechnology to flourish are targeting tumors, elevated bioavailability, and reduced cytotoxicity (Sanna V et al., 2012). Achieving specificity toward target cells, diminishing systemic toxicity, and attaining *in vivo* stability are some of the limitations that are considered while choosing nano delivery systems for the treatment of prostate cancer. To address the limitations, the budding application of aptamer-based targeted nano delivery and extracellular-mediated vesicle-mediated drug delivery turned out to be a very successful option. In addition to the specific delivery of siRNA and miRNA to cancer cells to achieve genetic silencing, which is a part of RNA nanotechnology, this could lead to efficient inhibition of prostate cancer. Aptamers are typically single-stranded oligonucleotides of 20–60 nucleotide length, possessing the capacity to bind to different molecules with specificity. Aptamers act like antibodies with the advantage of easier production methods at a low cost. A range of aptamer-based nano-carrier has been constructed for the effective delivery of drugs for the treatment of prostate cancer which enhanced tumor targeting. One of the initial aptamer-based nano delivery methodologies, PSMA aptamer/polo-like kinase 1(Plk1)-siRNA (A10-Plk1) chimera was established, which inhibited the growth of a prostate tumor. Thereafter, the second generation of PSMA-Plk1 chimeras was established to augment specificity and gene-specific silencing, thereby facilitating the upliftment of *in vivo* kinetics (Thakur A et al., 2021). Most significantly, it was observed that the second-generation chimeras restrained the growth and proliferation of prostate cancer cells at a lower concentration than the first generation. To date, 16 clinical trials of the use of nanoparticles in PC treatment were registered, of which five were completed, four terminated, one withdrawn, one active but not recruiting, and 5 still recruiting (gov 2022).

## 6 Conclusion

Continual progress in the field of prostate cancer diagnosis and treatment has enhanced clinicians’ capacity to grade patients by risk and prescribe medication based on cancer prognosis and patient preference. When compared to androgen restriction therapy, the first chemotherapy treatment can improve survival. Men with metastatic prostate cancer who are resistant to standard hormone therapy may benefit from abiraterone, enzalutamide, and other medicines. Methodologies involving nanomedicines exemplify significant advancements in the drug delivery research arena. In terms of potential or utility, the design and functionality of NP vary greatly. It is worth noting that improving the selectivity of an NP-based drug delivery system can be performed by surface engineering of a specific NP of interest. The selection of a suitable surface marker, on the other hand, is critical for targeted NP-based therapeutic delivery. Overall, nanotechnology-based drug delivery has been extremely fruitful for cancer therapy, including PC treatment, with numerous advantages (such as passive tumor accumulation, active tumor targeting, and transport across tissue barriers) and drawbacks (such as toxicity and organ damage).
